# The impact of internet use on old-age support patterns of middle-aged and older adults

**DOI:** 10.3389/fpubh.2022.1059346

**Published:** 2023-01-13

**Authors:** Jingyi Wang, Lifei Gao, Guojun Wang, Baibao Hu

**Affiliations:** ^1^School of Insurance and Economics, University of International Business and Economics, Beijing, China; ^2^School of Economics, Beijing Technology and Business University, Beijing, China; ^3^Sunshine Life Insurance Corporation Limited, Beijing, China

**Keywords:** internet use, commercial pension insurance, formal care, independent pattern of old-age support, information access, social interaction

## Abstract

**Background:**

The trend towards low fertility and low mortality is prominant worldwide. The accelerating ageing and the pressure on public pensions are making the “dependent pattern of old-age support”, which relies on family and government, unsustainable. It is urgent for people to change their mindset about ageing and to develop a sense of “relying on themselves for oldage support”.

**Methods:**

This study incorporates the commercial pension insurance, formal care and the attitude towards independent old-age support pattern into the framework of “independent pattern of old-age support”, using the probit regression model and instrumental variables approach to examine the impact of internet use on old-age support patterns of middleaged and older people based on the CGSS 2012–2018 five-period data.

**Results:**

The more frequent internet use increased the likelihood of purchasing commercial pension insurance, accepting formal care, and endorsing independence in later life. The internet can promote the acceptance of independent pattern of old-age support by delivering information and facilitating social interaction.

**Discussion:**

Consistent with previous research, this study finds that internet use can promote the purchase of commercial pension insurance. There are no relevant studies on the impact of internet use on formal care and attitude towards independent old-age support pattern. Our finding provides important empirical and theoretical references for ageing countries to further transform old-age support patterns.

## Introduction

Humanity is entering an era of longevity characterised by low mortality and low fertility. As ageing and childlessness are increasing, there is a growing concern about the old-age support pattern. Reliance on the family has long been the dominant pattern of ageing. In East Asian cultural circles, the parent-child relationship is at the heart of the family relationship, with children taking the main responsibility for providing old-age support for their parents (1). Spousal relationships are valued more in Europe and America. Older people in these areas generally prefer living with their spouse for mutual care (2), followed by their adult children (3). However, with modernisation and the replacement of the traditional extended family by the nuclear family, older people can no longer rely solely on their families (4). Since World War II, the world has established social security system for the older adults to provide them financial security with the help of the state. However, as the global economy stagnated in the 1970s and the population became increasingly ageing, the sustainability of social security systems was threatened in many countries. The World Economic Forum report 2017 projected that without adjustments to existing pension systems, six countries—Japan, the United States, the United Kingdom, the Netherlands, Canada, and Australia—would have a $224 trillion shortfall in pension savings by 2050 (5). Hence, reliance on the government for old-age support also becomes unviable. Thus, the “dependent pattern of old-age support”, which relies on family and government, is inadequate to cope with the current situation, and it is urgent to transform the old-age support pattern.

The “independent and dependent pattern of old-age supports” are mutually contradictory. Independent pattern of old-age support emphasises the independence of the individual in accessing support in old age. One's personal savings and investments ensure financial support for old age, making the person financially independent. Commercial pension insurance represents a financially independent pattern of old-age support. The pension funds that individuals obtain through commercial pension insurance are essentially their own investments. Unlike the pension, instrumental support such as daily care cannot be provided independently; however, different styles of care can reflect a person's mental independence. The older people's acceptance of care from a non-family member shows their mental independence from family members. Therefore, receiving formal care services in a nursing home or in the community from professional caregivers indicates a mentally independent pattern of old-age support.

Multiple measures were adopted worldwide in recent years to promote a change from dependent to independent pattern of old-age support. The establishment of the Individual Retirement Account in the United States, the Central Provident Fund system in Singapore, and the privatisation of pensions in Chile are initiatives to develop commercial pension insurance. Countries such as Germany, Japan, South Korea, and China have implemented long-term care insurance systems, providing policy guarantees for older people to receive formal care services. However, due to historical factors and cultural traditions, the independent pattern of old-age support is not popular. Among the Organisation for Economic Co-operation and Development countries where private pensions are voluntary, the Czech Republic, Germany, New Zealand, and the United States have the highest rates of private pension coverage, but only at around 50%. In other countries, private pension coverage remains at around 20%, which is a limited complement to public pensions (6). The South Korean conviction in filial responsibility has led to a negative view of institutional care, with 86% of South Koreans believing that frail older people should be cared for by adult children (7). The transition to independent pattern of old-age support requires national policies to create a supportive external environment. People's acceptance is key to an optimised old-age support pattern. It is therefore a common concern worldwide to promote better acceptance of independent pattern of old-age support and to address the current inadequacy of support in old age due to ageing and infantilisation.

The use of the internet in the information age has accelerated the transition of old-age support pattern. In China, as of December 2020, the number of internet users reached 989 million and the internet penetration rate reached 70.4% (8). With the increase in its penetration, the internet has gradually become the main way for people to access information. As the current mainstream communication medium, the internet is characterised by its abundance, rapidity, and interactivity in disseminating information (9). This has caused a profound impact on traditional lifestyles as well as mindsets and has subliminally changed people's choices of old-age support pattern. Existing research suggests that the use of the internet can promote the purchase of commercial pension insurance and increase the independence of individuals in preparing their pension funds (10, 11). However, the impact of internet use on independent pattern of old-age support from other perspectives remain unexplored. It is important to analyse the impact of internet use on the independent pattern of old-age support, which enable us to explore measures to help people live more independently in later life.

In China, family members are still the main source of financial, instrumental, and emotional support for older people (12, 13). However, rapid ageing and declining fertility rates are preventing older people from receiving adequate support from their families. Public pensions, long-term care, and other social services remain inadequate to meet their needs (14). The Chinese government has taken several measures to this end, such as implementing tax-deferred commercial pension insurance and launching a nationwide pilot long-term care insurance system, creating little effect. Lacking knowledge and experience, many Chinese people are still unaware of or unfamiliar with the private pension plan (15). In terms of acceptance of formal care, a survey based on rural older people showed that only 10.8 and 8.5% of older population were willing to go to institutions and community-based care (16). This study takes China, a representative country, as the subject of this research and incorporates commercial pension insurance, formal care, and the attitude towards independent old-age support pattern into the framework of “independent pattern of old-age support”. By examining whether and how the use of the internet has influenced the acceptance of independent pattern of old-age support, this study aims to identify effective ways to promote the transformation of the old-age support pattern in China and to provide references for ageing countries worldwide that are facing similar problems.

The remainder of this study is organised as follows. The theory and literature review section gives the theoretical origins of the concept of “independent pattern of old-age support” and reviews the literature on internet use and ageing. The data, variables, and descriptive statistics section describes the data sources and variable selection and performs preliminary descriptive statistical analysis. The methods section describes the measures used in this study. The results section presents the baseline regression results, tests the robustness of the results using a variety of methods, and explores the channels. Finally, the research findings and its relevant discussions, research contributions, further research directions, and policy recommendations are summarised.

## Theory and literature review

### Independent pattern of old-age support in active ageing

The independence of older people is often mentioned while discussing active ageing. Active ageing is a scientific concept developed in response to the challenges posed by population ageing, emphasising the various positive conditions of old age throughout the ageing process, such as physical health, positive emotional states, social participation, and security (17). “Independence” takes many forms in active ageing. Valuing self-reliance, independence, and personal responsibility is a key theme in New Zealand's implementation of the Active Ageing Strategy. Independence here is a financial concept suggesting that people should be responsible for their personal growth and development and be responsible in financing their own retirement and living a healthy life (18). Additionally, active ageing is concerned with physical functional independence and mental independence. One of the goals of Ageing in Place, an important strand of active ageing, is to explore technology's utilisation to ensure that older people can achieve independent living when they become disabled, rather than relying on others for care (19). Another important branch of active ageing, successful ageing, emphasises emotional and spiritual adjustment and autonomy, arguing that autonomy and independence can reduce stress and provide purpose and meaning while facing illness (20, 21).

As representatives of financial and mental independence, the purchase of commercial pension insurance and the choice of formal care both reflect active ageing. Apart from enabling older people to access social services such as healthcare, the purchase of commercial pension insurance also enhances their awareness and ability to plan and manage their finances, increasing their financial security, which is crucial to achieving active ageing (22). Receiving formal care in a nursing home or in the community allows older people to receive support and services when there is insufficient support from their children. Choosing formal care also means that older people are less dependent on their children, have a greater ability to maintain social relationships, and could receive emotional support from multiple sources beyond their children (22).

### How the internet influences people's views and behaviour

Early media impact studies focused on the impact of the information disseminated by the media on users, such as cultivation theory, which suggests that the content presented by the communication media in modern society can influence people's perception and understanding of the real world to some extent (23). It originated in the study of the television effect and was later extended to a range of other forms of media (24, 25). The internet is currently an extensively used medium. Contrary to traditional media, the internet offers an increasingly interactive media environment that allows users to choose their own media content. This increasingly prominent personalisation would change the way the media influence users (9). However, the internet has not changed the basic principles of cultivation theory (26). The impact of the abundant information available on the internet on users' attitudes and beliefs may increase (27). Personalisation may also increase the cognitive and emotional engagement of media users, which in turn enhances the effect of the internet on people (28). Some studies have shown that the constant political polarisation of views received from extremists on the internet can decrease the political trust among citizens (29). A study on Indian teenagers shows that those who use internet as their main source of information have a highly negative attitude towards Muslims and Islam, which is closely linked to the negative reports of the same in the Western media on internet (30).

Additionally, the highly interactive nature of the internet allows it to affect people's views and behaviour by influencing interpersonal and group interactions. Social information processing theory suggests that two parties communicating online could increase their intimacy through creative use of verbal cues and interaction strategies (31). The internet-enhanced self-representation hypothesis suggests that the internet is perceived as a safe place and therefore people tend to reveal more information to their friends online (32). It allows for closer contact and more frequent interaction between online communicators and enhances offline interaction to a certain extent. Internet offers the opportunity to develop new offline relationships. Some relationships may start online, but usually continue offline or in a mixed media form (33). There is also evidence suggesting that frequent internet users participate in informal social activities and in various voluntary organisations more frequently than others (34). In addition, frequent internet use is positively correlated with community engagement. Members with access to the internet are more engaged and active in the community (35). Herd behaviour can arise naturally when the strength of exogenous social interactions is high (36). Thus, we find that people who interact with each other regularly tend to think and behave similarly (37). Internet has enabled easier communication among people, which can increase the frequency and intensity of herd behaviour and thus have an impact on social attitudes and behaviour.

### Internet use, active ageing, and independent pattern of old-age support

Internet use can promote active ageing. As an information medium, older people can obtain health-related knowledge from the internet, thus helping them to maintain the functional literacy skills required to manage health (38, 39). As an important facilitator of interpersonal and group interactions, older people can interact online to build mutually supportive relationships and strengthen ties with family and friends through the internet, thereby reducing people's depressed isolation and increasing perceived social support and connectedness (40, 41). Internet access seems to improve connexions with the outside world, increase the ability and willingness to leave home, and increase the desire to meet others. The internet also offers older people the possibility of participating in diverse activities and increases the frequency of their participation in leisure activities (42, 43).

While the internet promotes active ageing, it is also supposed to increase the acceptance of independent pattern of old-age support, which is an important manifestation of active ageing. Access to insurance information is an important influencing factor in the purchase of commercial pension insurance. Professional, accurate, and comprehensive insurance information on the internet can raise people's awareness and promote the purchase of commercial pension insurance (10, 11). Additionally, people can obtain relevant explicit or implicit information through social interactions (11), and the internet has a facilitating effect on social interactions, thus further promoting the purchase of commercial pension insurance. However, different information on the internet has different effects on the purchase of commercial pension insurance. Positive messages on news portals can encourage people to buy commercial (pension) insurance; however, BBS messages about insurance are usually negative and therefore have a deterrent effect on commercial (pension) insurance participation (44). No evidence currently suggests that internet use promotes the choice of formal care, but studies have shown that the more the older people know about nursing homes, the more likely they are to choose institutional care (45). The information-mediating properties of the internet and its facilitation of social interaction can provide people with relevant information and promote their understanding of formal care. Access to adequate social support from family and friends and participation in various activities means that relatively more social resources are available to older people, which can increase their sense of security and independence, thus reducing their dependence on their children in old age and increasing their propensity to choose institutional care (46). Support from family and friends as well as opportunities to get involved in social activities can be obtained from the internet. From these perspectives, internet use can increase the acceptance of formal care. However, there are also arguments that individuals with limited family solidarity may have relatively low expectations of their family as a potential source of care and may be more receptive to using nursing homes (47). The internet's facilitation of family's and friend's contact may therefore also dampen the demand for formal care and have a debilitating effect on its acceptance. Hence, the impact of the internet on independent pattern of old-age support is uncertain, and its ultimate effect must be further explored.

## Methods

### Data and samples

The data are obtained from the Chinese General Social Survey (CGSS) database. China Survey and Data Centre of Renmin University of China surveyed the data. Based on the principle of Probability Proportionate to Size Sampling, 100 county-level units and 5 metropolitan areas, 480 village/residential committees and 12,000 individuals are sampled nationwide in each survey year to collect tracking information on social change trends in urban and rural areas, with data covering 28 provincial administrative units. The CGSS collects information at multiple levels—social, community, family, and individual—with a section specifically asking respondents about their use of the internet and providing a range of information about old-age support patterns in relation to this study. We select people aged 45 and over as the sample based on World Health Organization's definition of the middle-aged and older adults (48). Using five periods of CGSS data from 2012, 2013, 2015, 2017, and 2018, a database containing 31,049 samples is created after removing missing and outlier samples, of which 2012, 2013, 2015, 2017, and 2018 contain 5,700, 5,152, 5,817, 7,472, and 6,908 samples, respectively. It should be noted that the question on formal care only appeared in the 2017 questionnaire and was asked of a random sample of respondents from the total sample. Therefore, we only obtained 2,436 one-year data on this issue.

### Variables

#### Dependent variables

The dependent variable in this study is the individual's acceptance of independent pattern of old-age support. In the CGSS questionnaire, the question “Do you have commercial pension insurance” reflects the respondents' acceptance of financially independent pattern of old-age support. The variable takes a value of 1 if the respondent possesses commercial pension insurance, otherwise takes a value of 0. The question “Who do you think should primarily take care of the elderly in our country?” asks respondents' acceptance of formal care, which is a mentally independent pattern of old-age support. This variable takes the value of 1 if the respondent selected “government, private business/for-profit, non-profit/charity or religious organization” and 0 if the respondent selected “family, relatives or friends”. In addition to commercial pension insurance and formal care, the CGSS asks about respondents' attitude towards independent old-age support pattern: “Who do you think should be primarily responsible for the old age of elderly people with children?” The option “by the children or the government” represents the dependent pattern of old-age support, while the option “by themselves” represents the independent pattern of old-age support. This variable takes the value one, if the respondent believes that they should be responsible, or 0 if the respondent believes that their children or the government should be responsible. Ultimately, we construct three binary variables “commercial pension insurance”, “formal care”, and “attitude towards independent old-age support pattern” to indicate whether an individual accepts independent pattern of old-age support.

#### Independent variables

The independent variable in this study is internet use. Referring to previous studies (49, 50), we use “past year's use of the internet” to reflect the frequency of individual internet use. This variable classifies the frequency of internet use into five levels: “never”, “rarely”, “sometimes”, “often”, and “very often”, each with a value from 1 to 5, wherein higher level indicates more frequent use of the internet.

#### Control variables

Based on previous studies (11, 46, 51) on the factors influencing the old-age support patterns, we select gender (male = 1; female = 0), age, household registration (agricultural = 1; non-agricultural = 0), education, logarithm of per capita household income (income), number of properties (house), and self-rated health (SRH) as control variables at the level of individual characteristics. Where SRH is an integer variable with a value between 1 and 5, wherein higher value indicates better health status. Marital status (married = 1; unmarried, divorced or widowed = 0) and number of children are chosen as control variables at the household characteristics level. The coverage of basic medical insurance (yes = 1; no = 0) and basic pension insurance (yes = 1; no = 0) are added as control variables at the level of social characteristics.

### Descriptive statistics

The independent variable in this study is a continuous variable with values ranging from 1 to 5. The higher the value, the more frequent the internet use is, and the never-used value is one. This section divides the sample into those who use the internet and those who do not, based on whether the frequency of internet use is greater than one. By conducting descriptive statistics on different groups, we initially explore the influence of the frequency of internet use on independent pattern of old-age support. The results of the descriptive statistics are shown in Table 1.

**Table 1:** Descriptive statistics results.

**Variables**	**Mean**			
	**Total (1)**	**Internet users (2)**	**Non-Internet users (3)**	**Mean difference (4)**
Commercial pension insurance	0.054	0.111	0.028	−0.084^***^
Formal care	0.353	0.428	0.305	−0.123^***^
Attitude towards independent old-age support pattern	0.407	0.470	0.378	−0.092^***^
Age	60.476	55.836	62.639	6.803^***^
Gender	0.498	0.520	0.488	−0.032^***^
Household registration	0.603	0.379	0.707	0.328^***^
Education	2.440	3.005	2.177	−0.828^***^
Income	9.323	10.052	8.984	−1.068^***^
House	1.106	1.161	1.080	−0.081^***^
SRH	3.301	3.597	3.163	−0.434^***^
Marital	0.819	0.876	0.792	−0.084^***^
Child number	2.095	1.486	2.379	0.893^***^
Basic pension insurance	0.781	0.844	0.752	−0.092^***^
Basic medical insurance	0.931	0.945	0.925	−0.020^***^

*T*-statistic value in parentheses.

*** *p* < 0.01, ^**^p < 0.05, ^*^p < 0.1.

Table 1 shows the differences in sample means between the variables for the full sample, internet users, and non-users. According to columns (1–3), in terms of commercial pension insurance, the proportion of people in the whole sample who have purchased commercial pension insurance is higher than non-internet users and lower than internet users. In terms of formal care, the proportion of people accepting formal care in the overall sample is higher than non-users and lower than internet users. In terms of attitude towards independent old-age support pattern, the proportion of people in the overall sample who believe that they should be responsible for their own ageing is higher than non-users and lower than internet users. As shown in column (4), the proportion of the internet users who accepts independent pattern of old-age support is significantly higher than that of non-users. From the descriptive statistics, we can tentatively infer that the more frequently the internet is used, the more likely people are to embrace the independent pattern of old-age support. However, the acceptance of independent pattern of old-age support is influenced by many factors and whether there is a significant relationship between the frequency of internet use and the acceptance of independent pattern of old-age support must be confirmed by further empirical analysis.

### Probit regression model construction

As the three dependent variables in this study are binary variables, a probit regression model is chosen to analyse the impact of the internet use on the independent pattern of old-age support. The specific expressions are as follows.


(1) $$\begin{array}{l} \text{Pr}(Commercial\;pension\;insurance_{i}=1|X_{i})=\emptyset (\alpha_{0}+\alpha_{1}int_{i}\\ \;+\sum \alpha_{j}Control_{ij}+\varepsilon_{i}) \end{array}$$

(2) $$\begin{array}{l} \text{Pr}(Formal\;care_{i}=1|X_{i})=\emptyset (\beta_{0}+\beta_{1}int_{i}\\ \;+\sum \beta_{j}Control_{ij}+\varepsilon_{i}) \end{array}$$

(3) $$\begin{array}{l} \text{Pr}(attitude_{i}=1|X_{i})=\emptyset (\gamma_{0}+\gamma_{1}int_{i}\\ \;+\sum \gamma_{j}Control_{ij}+\varepsilon_{i}) \end{array}$$

Among them, *Commercial pension insurance*_*i*_, *Formal care*_*i*_, and *attitude*_*i*_ are three dependent variables, which represent whether individuals have purchased commercial pension insurance, whether they accept formal care, and whether they believe that people should be responsible for their own ageing. *X*_*i*_ represents all variables that can affect the independent pattern of old-age support. *int*_*i*_ is the independent variable in this manuscript, representing the frequency of internet use of individual i. *Control*_*ij*_ is a set of control variables including the respondent's gender, age, household registration, education level, household income per capita, health status, marital status, number of children, social security possession, and year of interview. α_1_β_1_γ_1_ are the results of interest, reflecting the extent and direction of the impact of internet use on independent pattern of old-age support. ε_*i*_ is the random error term.

## Results

### Baseline regression results

Table 2 demonstrates the specific impact of internet use on the independent pattern of old-age support. Columns (1, 2) show the impact of internet use on commercial pension insurance, columns (3, 4) show the impact of internet use on formal care, and columns (5, 6) show the impact of internet use on attitude towards independent old-age support pattern. The frequency of internet use has a positive and significant effect on the acceptance independent pattern of old-age support. After calculating the marginal effects corresponding to each coefficient, the results show that for every 1 percentage point increase in the frequency of internet use, without controlling for the relevant variables, the probability of purchasing commercial pension insurance, the probability of accepting formal care, and the probability of believing that people should be responsible for their own ageing will increase by 2.3, 4.2, and 3.1 percentage points, respectively. After controlling for relevant variables, for every 1 percentage point increase in the frequency of internet use, the probability of purchasing commercial pension insurance, the probability of accepting formal care, and the probability of believing that people should be responsible for their own ageing will increase by 1.1, 3.2, and 1.2 percentage points, respectively. The results show that more frequent internet use by people may increase their acceptability of the independent pattern of old-age support.

**Table 2:** Impact of internet use on independent pattern of old-age support.

**Variables**	**Commercial pension insurance**		**Formal care**		**Attitude towards independent old-age support pattern**	
	**(1)**	**(2)**	**(3)**	**(4)**	**(5)**	**(6)**
Internet use	0.228^***^	0.116^***^	0.115^***^	0.090^***^	0.080^***^	0.032^***^
	(31.103)	(12.009)	(6.735)	(4.130)	(15.407)	(4.945)
Age		−0.008^***^		0.018^***^		0.012^***^
		(−5.216)		(5.239)		(13.158)
Gender		−0.022		−0.008		−0.091^***^
		(−0.843)		(−0.140)		(−6.002)
Household registration		−0.267^***^		−0.329^***^		−0.257^***^
		(−8.110)		(−4.747)		(−13.989)
Education		0.144^***^		0.046		0.104^***^
		(7.006)		(1.125)		(9.700)
Income		0.071^***^		0.012		0.010^**^
		(5.303)		(0.940)		(2.254)
House		0.167^***^		−0.170^***^		−0.019
		(9.362)		(−3.410)		(−1.418)
SRH		0.083^***^		−0.014		−0.009
		(6.477)		(−0.545)		(−1.264)
Marital		0.025		−0.012		0.084^***^
		(0.662)		(−0.164)		(4.186)
Child number		−0.005		−0.100^***^		−0.050^***^
		(−0.385)		(−2.614)		(−6.752)
Basic pension insurance		−0.064^*^		−0.063		0.073^***^
		(−1.887)		(−0.869)		(3.851)
Basic medical insurance		−0.216^***^		−0.069		0.008
		(−4.392)		(−0.631)		(0.276)
Year		0.005				−0.009^***^
		(0.874)				(−2.687)
Constant	−2.112^***^	−13.209	−0.622^***^	−1.161^***^	−0.383^***^	17.272^**^
	(−97.540)	(−1.087)	(−13.770)	(−3.840)	(−31.842)	(2.525)
Obs	31,049	31,049	2,436	2,436	31,049	31,049
R square	0.069	0.114	0.014	0.057	0.006	0.030

*T*-statistic value in parentheses.

*** *p* < 0.01,

** *p* < 0.05,

* *p* < 0.1.

### Further analysis

#### Endogeneity tests

The relationship between individual internet use and the acceptance of independent pattern of old-age support may be endogenous due to omitted variables and two-way causation. First, difficult-to-measure variables such as respondents' personalities, habits, or ability to accept new things may influence both individual internet use and acceptance of independent pattern of old-age support, thus creating the problem of omitted variables. Second, individuals who embrace independent pattern of old-age support may already have less social support from friends and family offline and therefore must rely on the internet to alleviate their isolation and use it more frequently, leading to a two-way causal problem. This study uses instrumental variables approach to test the endogeneity question.

Referring to the study by Wang, Nie, and Liu (50), we construct a provincial internet penetration variable using the share of internet access ports in the total population in the year surveyed as an instrumental variable for individual internet usage. Internet penetration can influence individual internet use, satisfying the relevance of the instrumental variable. However, internet penetration, as a macro-level figure, does not directly impact the acceptance of independent pattern of old-age support by micro-individuals in the current period. Additionally, referring to Yue, Wang, and Zhang (52), this study calculates the mean value of annual internet usage frequency for people under 45 years old in each province in the database as an additional instrumental variable for individual internet usage. We use a sample of middle-aged and older people aged 45 years and above. According to the peer effect theory, the frequency of internet use by young people can influence the frequency of internet use by middle-aged and older people. However, it has a relatively small impact on the acceptance of independent pattern of old-age support for middle-aged and older people. In addition, as a macro-level variable, this further attenuates the impact on individual's attitude towards old age, satisfying the conditions for selection of the instrumental variable. Table 3 shows the results of the first- and second-stage regressions when internet penetration and the mean value of internet use, are used as instrumental variables. The coefficients of the instrumental variables in the first stage regressions are significantly positive when internet penetration and internet usage mean are used as instrumental variables, and the F-statistic values are all >10, indicating that the instrumental variables have been selected effectively. The results of the second stage of the regression show that after the endogeneity problem is mitigated using instrumental variables, there is still a significant positive relationship between frequency of internet use and independent pattern of old-age support. This is consistent with the findings of the baseline regression.

**Table 3:** Endogeneity test results.

**Variables**	**Commercial pension insurance**		**Formal care**		**Attitude towards independent old-age support pattern**	
	**First stage**	**Second stage**	**First stage**	**Second stage**	**First stage**	**Second stage**
Internet use		0.439^***^		0.470^**^		0.225^***^
		(5.114)		(2.496)		(4.198)
Internet penetration	1.087^***^		1.116^***^		1.087^***^	
	(19.546)		(6.063)		(19.546)	
Control	Yes	Yes	Yes	Yes	Yes	Yes
Phase I *F*-test	1,304.55		127.49		1,304.55	
Wald test		14.8		4.63		13.55
Obs	31,049		2,436		31,049	
	Private pension		Formal care		Attitude towards independent old-age support pattern	
	**First stage**	**Second stage**	**First stage**	**Second stage**	**First stage**	**Second stage**
Internet use		0.663^***^		0.341^***^		0.322^***^
		(6.862)		(2.702)		(5.697)
Internet Use_mean	0.284^***^		0.621^***^		0.284^***^	
	(20.520)		(9.145)		(20.520)	
Control	Yes	Yes	Yes	Yes	Yes	Yes
Phase I F-test	1,300.92		133.65		1,300.92	
Wald test		35.03		4.30		28.44
Obs	31,049		2,436		31,049	

*T*-statistic value in parentheses.

*** *p* < 0.01,

** *p* < 0.05,

^*^p < 0.1.

#### Robustness tests

##### Replace independent variables

The baseline regression section uses internet usage frequency to represent an individual's internet usage. The indicator can cover the CGSS sample for all years 2012–2018. Additionally, the CGSS questionnaires for 2017 and 2018 include a series of questions on individuals' internet use from different perspectives. This section therefore tests the robustness of the baseline regression results by replacing the measure of the independent variable “internet use”. The independent variables “minutes of internet use” and “internet use or not” are constructed from the question “In the past 12 months, how much time did you usually spend on the internet *via* a computer or various mobile applications each day?” and the question “In the last 6 months, did you go online?” The minutes of internet use is the logarithm of the number of minutes spent online per day. Internet use or not is a binary variable, taking a value of 1 if the internet is used and 0 otherwise. Table 4 shows the regression results after replacing the independent variables. The longer the people use the internet, the more likely they are to accept independent pattern of old-age support. Those who use the internet are more likely to embrace independent pattern of old-age support than those who do not. This is similar to the baseline results.

**Table 4:** The results of replacing independent variables.

**Variables**	**Commercial pension insurance**		**Formal care**		**Attitude towards independent old-age support pattern**	
	**(1)**	**(2)**	**(3)**	**(4)**	**(5)**	**(6)**
Minutes of internet use	0.055^***^		0.055^***^		0.029^***^	
	(5.739)		(3.484)		(4.643)	
Internet use or not		0.212^***^		0.193^***^		0.110^***^
		(4.772)		(2.798)		(3.995)
Control	Yes	Yes	Yes	Yes	Yes	Yes
Obs	14,380	14,380	2,436	2,436	14,380	14,380
R square	0.111	0.109	0.056	0.055	0.032	0.032

*T*-statistic value in parentheses.

*** *p* < 0.01, ^**^p < 0.05, ^*^p < 0.1.

##### Replace database

In this section, a replacement database is chosen to test the robustness of the baseline regression results. The China Longitudinal Ageing Social Survey (CLASS) is a survey targeting Chinese people aged 60 and older. It systematically collects data on the social and economic background of China's older adult population, including their use of the internet and their attitude to independent pattern of old-age support. In terms of internet use, CLASS asks respondents about their use of the internet in the past 3 months, using a scale of 1–5 ranging from never to always. Regarding independent pattern of old-age support, CLASS asks respondents about their purchase of commercial pension insurance and their acceptance of formal care. For commercial pension insurance, as with the baseline regression, the value is taken as 1 if the respondent possesses commercial pension insurance, otherwise it is taken as 0. In terms of formal care, the question “Where do you plan to spend most of your time in your old age?” reflects respondents' attitude towards formal care. A value 1 is assigned if the respondent chooses to live in a community day-care station or a nursing home, because this option indicates a high level of acceptance of formal care. If the respondent chooses to live in their own home or in their children's home, which indicates a low level of acceptance of formal care, the variable takes the value of 0. The selection of control variables and the empirical methodology remains consistent with the baseline regression. Table 5 shows the regression results after replacing the database. There is still a significant positive relationship between internet use and independent pattern of old-age support after changing databases. The more frequently people use the internet, the more likely they are to embrace independent pattern of old-age support. This further supports the robustness of the basic regression results.

**Table 5:** The results of replacing the database.

**Variables**	**Commercial pension insurance**		**Formal care**	
	**(1)**	**(2)**	**(3)**	**(4)**
Internet use	0.221^***^	0.146^***^	0.230^***^	0.174^***^
	(12.368)	(6.672)	(16.941)	(10.935)
Control	No	Yes	No	Yes
Obs	11,281	11,281	11,281	11,281
R square	0.062	0.100	0.051	0.088

*T*-statistic value in parentheses.

*** *p* < 0.01, ^**^p < 0.05, ^*^p < 0.1.

#### Channel analysis

The previous results show that internet use can have an impact on independent pattern of old-age support. The more frequently people use the internet, the more likely they are to accept this pattern. What are the pathways through which internet use influences people's acceptance of independent pattern of old-age support? According to cultivation theory, the internet can significantly influence people's perception and understanding of the real world through the information it conveys (23). Additionally, the internet's facilitation of social interaction has led to an increase in the frequency and intensity of herd behaviour (36), further influencing human thoughts and behaviour.

To verify the existence of the two channels, we construct two channel variables, information access and social interaction. The two channel variables are first replaced as dependent variables in the baseline regression to explore the impact of internet use on information access and social interaction. Then they are put into the baseline regression. If the channel makes sense, the following two conditions should be met: 1. Internet use is able to have a significant effect on both channel variables; 2. The coefficients on the channel variables are significant and as expected when the two channel variables are included in the baseline regression, and the main independent variable, internet use, is less significant or its coefficient is significantly smaller (53).

Information access is a binary variable in this study. It takes the value of 1 if the respondent mainly obtains information from the internet, otherwise it takes the value of 0. This variable captures the influence of the information conveyed by the internet on people. This is because people use the internet for various purposes, including study and work and social entertainment (54). Some people may not use the internet to access information. For these people, the information conveyed by the internet has limited impact on them. The influence of information on the internet is stronger for respondents, if their main source of information is the internet.

Contexts of interaction can be classified according to the purpose of the social interaction as work situations,^1^ social situations,^2^ and daily interactions with acquaintances (55). Based on this definition and the available data, we construct the channel variable of social interaction. This variable measures how often respondents shopped, participated in cultural activities, gathered with relatives, and met with friends during their free time in the past year. All the four activities are integer variables with values between 1 and 5, with higher values representing more frequent participation in the activity. After integration, social interactions are eventually constructed as continuous variables with values ranging from 4 to 20, with higher values being associated with higher frequency of social interactions.

Table 6 shows the results of the channel test. As shown in Table 6, the more frequently the internet is used, the more likely individuals are to obtain information from the internet and the more frequently they participate in social interactions. However, the two channels do not make sense in all cases. The coefficient on information access is significantly positive when the dependent variable is commercial pension insurance, however, insignificant when the dependent variables are formal care and attitude towards independent old-age support pattern. It suggests that access to information *via* the internet can facilitate the purchase of commercial pension insurance, however, does not increase the acceptance of formal care, and has a limited role in promoting an overall transformation in attitude towards old age. The internet's facilitation of social interactions can have a positive and significant effect on the three dependent variables, suggesting that the more frequently individuals participate in social interactions, the more likely they are to purchase commercial pension insurance, to accept formal care, and to agree that people should be independent in their old age. The results show that the internet can promote the purchase of commercial pension insurance through the information it conveys and the social interaction it facilitates. However, increased acceptance of formal care and a change in attitude towards independent old-age support pattern can only be achieved by promoting social interaction.

**Table 6:** Channel tests results.

**Variables**	**Information access**	**Commercial pension insurance**	**Formal care**	**Attitude towards independent old-age support pattern**
Internet use	0.761^***^	0.100^***^	0.077^***^	0.033^***^
	(66.597)	(8.471)	(2.908)	(4.017)
Information access		0.095^**^	0.081	−0.004
		(2.331)	(0.888)	(−0.123)
Control	Yes	Yes	Yes	Yes
Obs	31,049	31,049	2,436	31,049
R square	0.586	0.114	0.058	0.030
Variables	Social interaction	Commercial pension insurance	Formal care	Attitude towards independent old-age support pattern
Internet use	0.276^***^	0.106^***^	0.084^***^	0.030^***^
	(24.738)	(10.826)	(3.766)	(4.575)
Social interaction		0.039^***^	0.022^*^	0.008^**^
		(6.832)	(1.751)	(2.269)
Control	Yes	Yes	Yes	Yes
Obs	31,049	31,049	2,436	31,049
R square	0.171	0.117	0.058	0.030

*T*-statistic value in parentheses.

*** *p* < 0.01,

** *p* < 0.05,

* *p* < 0.1.

## Discussion

This study classifies old-age support patterns as dependent and independent patterns and for the first time incorporates commercial pension insurance, formal care, and attitude towards independent old-age support pattern into the framework of “independent pattern of old-age support”, using longitudinal data on a nationally representative sample in China to investigate whether the use of the internet can promote the acceptance of independent pattern of old-age support. We find that more frequent internet use increases the likelihood of people purchasing commercial pension insurance, accepting formal care, and agreeing that people should be independent in old age. The use of instrumental variables enhances the effect of causal inference, and the robustness of the results is demonstrated by adopting various robustness tests, leading to the conclusion that the internet increases the acceptance of independent pattern of old-age support. There have been studies that classify the patterns of old-age support according to the different providers of financial support and daily care, however, do not emphasise the independence in ageing. These studies are limited to the influence of personal endowments (47), intergenerational support, social security (51), and other factors (56) on the choice of old-age support pattern, and do not examine internet use. Shi Hao et al. conducted a study on the relationship between media use and commercial insurance and found that using the internet can promote the purchase of commercial insurance (57). But the commercial insurance here is commercial medical insurance and not include commercial pension insurance. Some studies have focused on the impact of internet use on the purchase of commercial pension insurance, while the impact of internet use on attitude to formal care and independent pattern of old-age support is unexplored. Wu, Yang, and Yin (10) as well as Wu, Bian, and Nie (11) found that internet use can facilitate people's purchase of commercial pension insurance, which is consistent with the findings of our study.

Additionally, the study focuses on the information-mediated identity and interactive nature of the internet and examines both channels of information access and social interaction at an empirical level. Internet can facilitate the purchase of commercial pension insurance through the information it conveys. People's perceptions can be influenced by the wide range of information available on the internet about commercial pension insurance. This information can motivate individuals to prepare more actively for ageing in a different manner and to increase their purchases of commercial pension insurance (10, 58). Although studies have shown that prior knowledge and familiarity are also important contributors to the use of formal care services (59), we find that information delivered by the internet has a limited impact on formal care acceptance. This may be because the decision to receive formal care involves an evaluation of the conditions of the place of care, caregivers, etc., and relevant information is inadequate in the internet. Social interaction enables more information to be obtained through verbal communication and observational learning (11, 60), complementing the information obtained from the internet. Our findings also suggest that the internet's enhanced effect on social interactions promotes the purchase of commercial pension insurance and increases the acceptance of formal care, prompting a shift towards relying on themselves in old age. As the internet allows for customised information, people who access information on independent pattern of old-age support *via* the internet are likely to be people who are already interested and have an open attitude towards it. Those who oppose independent pattern of old-age support may not follow the news about it at all. Therefore, they will not be influenced by the information conveyed through the internet, which will weaken our inferences about information access as a channel to some extent.

There are three limitations to this study. First, non-panel data prevent us from making more effective causal inferences by controlling for individual fixed effects. Second, the small amount of data available for formal care leaves a discrepancy in the persuasiveness of the results obtained compared to other results. Finally, in terms of influence channels, limited by data, we cannot consider the interference of the internet's personalised nature with information access as a channel. Additionally, the inventions using internet technology (40), the reduction of transaction costs by the internet, and the increased availability of insurance (61) are all potential pathways through which the use of the internet can influence the pattern of old-age support. Future studies could use analytics such as big data to produce more precise and specific analysis of the relationship between internet use and independent pattern of old-age support to generate more accurate results. Finding suitable variables to measure the impact of the internet on technological inventions, transaction costs, and insurance availability allows for a more diverse exploration of impact pathways.

Despite these limitations, this study has some theoretical and practical values. First, based on the definition of “independence” in the theory of active ageing, this study for the first time incorporates commercial pension insurance, formal care, and attitude towards independent old-age support pattern into the framework of “independent pattern of old-age support” and investigates the impact of internet use on the old-age support patterns of middle-aged and older people. Second, this study bridges the gap in existing research by demonstrating the impact of internet use on the acceptance of independent pattern of old-age support from different perspectives and exploring the channels. Finally, the findings demonstrate that the use of the internet can promote the acceptance of independent pattern of old-age support. This provides a practical direction for promoting rapid transition and optimisation of ageing and achieving active ageing in the current context of low acceptance of independent pattern of old-age support.

To make better use of the internet to facilitate the transformation of old-age support patterns, first, internet coverage and penetration should be ensured, especially in less developed areas and among the elderly. The government should accelerate the construction of digital infrastructure and increase subsidies for internet funding in poorer areas. Companies should develop user-friendly internet terminal devices to reduce barriers to internet use for older people. Equal, efficient and affordable internet access can help address the digital access divide. Second, the government and companies should focus on using the internet to propagate information about commercial pension insurance, formal care, and other related information to improve internet users' understanding of independent pattern of old-age support. Developing age-friendly digital financial products that are affordable and sustainable to meet the needs of older people. Third, while the society encourages older people to use the internet, it is also important for the elderly to learn to use the internet to obtain and exchange information with a positive, open and accommodating mindset. Fourth, internet regulation should be strengthened to avoid the negative impact of negative and inaccurate information on the acceptance of independent pattern of old-age support.

## Data availability statement

Publicly available datasets were analysed in this study. This data can be found here: http://cgss.ruc.edu.cn; http://class.ruc.edu.cn.

## Author contributions

JW: conception and design, writing—original draught, and preparation. LG and JW: methodology. LG, JW, GW, and BH: writing—review and editing. GW: funding acquisition. LG and GW: supervision. All authors contributed to the article and approved the submitted version.

## Footnotes

^1^These situations include shopping, business negotiations, work meetings, and attending classes.

^2^These situations include parties, balls, and outings.
